# Comorbidity profiles of patients experiencing homelessness: A latent class analysis

**DOI:** 10.1371/journal.pone.0268841

**Published:** 2022-05-24

**Authors:** Keshab Subedi, Shweta Ghimire

**Affiliations:** 1 Institute for Research on Equity and Community Health, ChristianaCare Health Systems, Wilmington, Delaware, United States of America; 2 Center for Bioinformatics and Computational Biology, University of Delaware, Newark, Delaware, United States of America; Bay Area Hospital, North Bend Medical Center, UNITED STATES

## Abstract

Individuals experiencing homelessness are known to have increased rates of healthcare utilization when compared to the average patient population, often attributed to their complex health care needs and under or untreated comorbid conditions. With increasing focus on hospital readmissions among acute care settings, a better understanding of these comorbidity patterns and their impacts on acute care utilization could help improve quality of care. This study aims to identify distinct comorbidity profiles of homeless patients, and to explore the correlates of the identified comorbidity profiles and their impact on hospital readmission. This is a retrospective analysis using electronic health records (EHR) of patients experiencing homelessness encountered in the hospitals of ChristianaCare from 2015 to 2019 (N = 3445). Latent class analysis (LCA) was used to identify the comorbidity profiles of homeless patients. The mean age of the study population was 44-year, and the majority were male (63%). The most prevalent comorbid conditions were tobacco use (77%), followed by depression (58%), drug use disorder (56%), anxiety disorder (50%), hypertension (44%), and alcohol use disorder (43%). The LCA model identified 4 comorbidity classes—“relatively healthy” class with 31% of the patients, “medically-comorbid with SUD” class with 15% of the patients, “substance use disorder (SUD)” class with 39%, and “Medically comorbid” class with 15% of the patients. The Kaplan-Meir curves of probability of readmission against time from the index visits were significantly different for the four classes (p<0.001). The multivariable Cox proportional hazard model adjusted for age, sex, race, ethnicity, and insurance type showed that the hazard for readmission among patients in medically comorbid with SUD class is 3.16 (CI: 2.72, 3.67) times higher than the patients in the relatively healthy class.

## Introduction

Homelessness is a serious national public health concern, with nearly 568,000 Americans experiencing homelessness on any given night as of 2019, and nearly 37% of these individuals unsheltered [1]. Homelessness is associated with an increased rate of morbidity and mortality, increased rates of healthcare utilization and costs, and poor health care outcomes [2–5]. When compared to the non-homeless patient population, much data has shown that individuals experiencing homelessness have higher rates of hospital readmission [6–8]. Much of the explanation for this has focused on unmet complex health care needs often aggravated by multiple comorbid conditions, including substance use disorders and mental health disease [3, 8, 9]. These conditions have been investigated individually as risk factors for a patients’ likelihood of developing homelessness [10, 11], and also as confounding factors for health care utilization patterns and outcomes of people experiencing homelessness [12–14]. However, despite much focus on the differences in readmission rates and medical conditions of those experiencing homelessness versus those who do not, little attention has been placed on whether any variation exists within the group of patients experiencing homelessness. There is limited data on the potential heterogeneity in the comorbidity patterns of homeless patients and its impacts on healthcare utilization and outcomes. Latent class analysis (LCA), a person-centered, finite mixture modeling approach allows investigation of such heterogeneity by identifying unobserved subgroups based on a vector of indicators [15]. The LCA model enables us to holistically explore the comorbidity profiles by taking multiple comorbidities as the model indicators. This study aims to identify distinct comorbidity profiles of patients experiencing homelessness, to explore correlates of identified comorbidity profiles, and to examine any variation in healthcare utilization patterns among these comorbidity classes. The study employs LCA to identify the comorbidity profiles of patients encountered in the hospitals of ChristianaCare. In doing so, the study further describes patterns of co-occurring disease among subtypes of patients experiencing homelessness and describe differences in healthcare utilization among these patterns. Understanding of the clustering patterns of comorbidities and their impact on readmissions could help health care providers and policy makers devise tailored interventions aimed at improving healthcare outcomes and reducing the acute care utilization burden among patients experiencing homelessness.

## Materials and methods

### Study design and setting

This is a retrospective analysis of electronic health records (EHR) of adults above age 18 identified as homeless during an acute care health encounter at ChristianaCare’s hospitals between January 1, 2015, and December 31, 2019. Encounters were restricted to emergency department visits and inpatient hospitalizations. We defined the first encounter within the study period as an index visit and we set follow up end date on December 31, 2020 to provide at least one year of follow up time for all the patients to record readmission related outcomes. Those visiting for an outpatient visit or who were admitted under “observation” status were excluded. ChristianaCare is one of the largest health care providers in the mid-Atlantic with a Level 1 trauma center serving all of Delaware and parts of Pennsylvania, Maryland, and New Jersey. The study was approved by ChristianaCare’s Institutional Review Board (IRB). The informed consent requirement was waived by the IRB.

### Data and data preparation

The analysis dataset was extracted from the EHR system of ChristianaCare. The patients experiencing homelessness were identified using one of three methods: having an ICD10 code Z59.0 associated with their encounter, a documented address matching a Delaware homeless shelter, or an encounter with social work or case management for homelessness. The demographic information in the data included patients’ age, gender, race, and ethnicity. We used Chronic Conditions Data Warehouse (CCW) Condition Algorithm to define chronic conditions of depression, tobacco use disorder, drug use disorder, hypertension, alcohol use disorder, chronic kidney disease (CKD), asthma, chronic obstructive pulmonary disease (COPD), hyperlipidemia, diabetes, obesity, schizophrenia, liver disease, viral hepatitis, epilepsy, post-traumatic stress disorder (PTSD), mobility impairment, ischemic heart disease, cancer, HIV AIDS, heart failure, atrial fibrillation, acute myocardial infraction, spinal cord injury, sensory deafness, intellectual disabilities. Comorbidities with prevalence of more than 75% (tobacco use), and less than 4% (acute myocardial infraction, spinal cord injury, sensory deafness, intellectual disabilities) were not included as indicator variable in the LCA analysis to ensure a meaningful separation of classes. The major outcome we analyzed was time to acute care readmission since the index visit.

### Statistical analysis

Summary statistics were calculated for patient’s demographic and clinical variables based on the index visit. For categorical variables, proportions were calculated and for continuous variables, mean and standard deviation are reported. Next, we employed latent class analysis (LCA) to identify the distinct comorbidity profiles of the patient population attributed with homelessness. LCA is a statistical method that generates homologous hidden typologies using a vector of observed variables. This is a “person-centered” approach of creating empirically derived typologies based on individual’s response to a set of observed indicator variables [15]. LCA helps understand unabsorbed within group heterogeneity in a population with respect to a given phenomenon and has been widely used in psychological and healthcare research. We fitted series of models with incremental class number starting with 2 classes. We used the fit indices AIC, BIC, LMR-LRT and model interpretability to decide the optimum number of classes. The assumption of conditional independence that the latent class explains all the shared variance among the observed variables in the model was evaluated using standardized residual z-score. Individuals were assigned to the class to which they had highest probability of membership. The class membership is summarized using conditional probability. The time-to-readmission from the index visit among the patients in four classes was evaluated using Kaplan-Meir curve and log-rank test. Further, a cox proportional hazard model was fitted with the comorbidity classes, age, gender, race, ethnicity, and insurance type as model covariates. The LCA was performed in Mplus 7.31 [16] and subsequent statistical analysis and visualization was done in R

## Results

### Characteristics of the study population

We identified 3445 unique patients attributed with homelessness who visited hospitals across ChristianaCare during the study period. The mean age of the study population was 44 years (SD = 15.6), and the majority were male (63%). The population was comprised of 52% White and 41% Black. Most were covered by Medicaid (65%), followed by Medicare (19%), whereas 11% did not have any insurance. The most prevalent comorbid conditions were tobacco use (77%), followed by depression (58%), drug use disorder (56%), anxiety disorder (50%), hypertension (44%), and alcohol use disorder (43%). Similarly, other comorbidities with prevalence of more than 15% are CKD (27%), asthma (25%), COPD (25%), hyperlipidemia (25%), diabetes (20%), obesity (17%), and liver disease (15%) (Table 1).

**Table 1:** Descriptive statistics of the study population. The values are count and percent unless otherwise noted.

Variable	All (n = 3445)
Age, mean (SD)	43.92 (15.62)
Sex	
Male	2179 (63.25)
Female	1265 (36.72)
Race	
Black	1425 (41.36)
White	1797 (52.16)
Other/unknown	223 (6.47)
Ethnicity	
Non-Hispanic or Latino	3114 (90.39)
Hispanic or Latino	197 (5.72)
Declined/Unknown	134 (3.89)
Insurance Type	
Commercial	148 (4.30%)
Medicaid	2232 (64.79%)
Medicare	684 (19.85%)
Self-Pay	381 (11.06%)
Comorbid conditions	
Tobacco use	2657 (77.13)
Depression	1992 (57.82)
Drug use disorder	1945 (56.46)
Hypertension	1512 (43.89)
Alcohol use disorder	1479 (42.93)
Chronic kidney disease (CKD)	922 (26.76)
Asthma	870 (25.25)
COPD	850 (24.67)
Hyperlipidemia	845 (24.53)
Diabetes	692 (20.09)
Obesity	601 (17.45)
Liver disease	519 (15.07)
Ischemic heart disease	441 (12.80)
Epilepsy	413 (11.99)
Post-traumatic stress disorder (PTSD)	388 (11.26)
Mobility impairment	176 (5.11)
Cancer	174 (5.05)
HIV/AIDS	147 (4.27)
Acute myocardial infraction	91 (2.64)

### LCA model selection and class description

**Fig 1:**
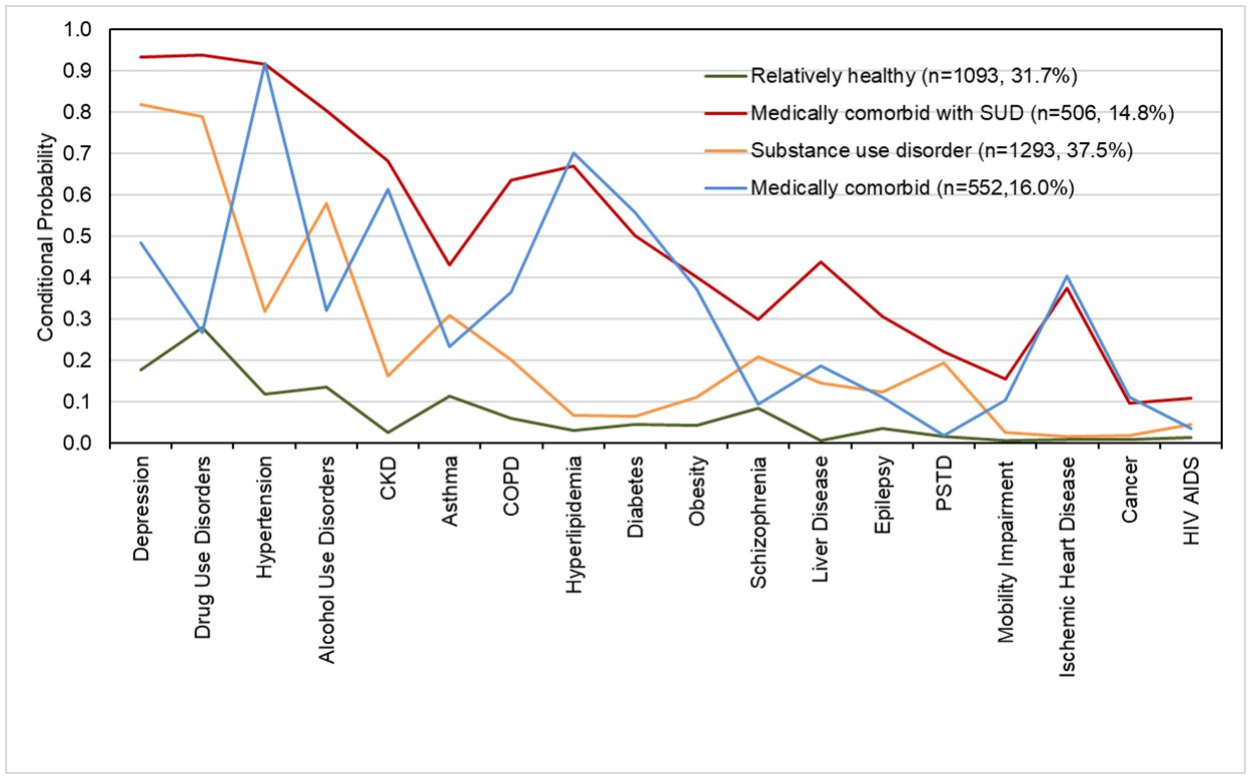
Conditional probabilities of individual comorbid conditions for four latent classes.

We found 4-class solution to be the best fitting based on fit indices and interpretability of the classes. The BIC, adjusted BIC, and AIC were lowest at 4-class solution. The distribution of patients in the four classes and conditional probabilities of each comorbid conditions for four latent classes is presented in Fig 1.

Class 1 included individuals with vey high probability for depression, drug use disorder, and alcohol use disorder. The individuals in this group have 82% probability of having depression and have probability of 79% and 58% for drug use disorder, and alcohol use disorder respectively. We labelled this class as “substance use disorder (SUD)”. The medical comorbidities with highest probability in this groups are hypertension (32%), followed by asthma (31%), and COPD (20%). The individuals in this class have low probability for other medical comorbidities. This is the largest class with around 38% of the patients. Among the four classes, this class has the lowest proportion of patients with racial identity of Black/African American (36%). Around 37% of the patients who fell in this group were female, and 76% were covered by Medicaid (Table 2).

**Table 2:** Summary of background characteristics of homeless patients in four latent classes. The values are count and percent unless otherwise noted.

Variable	Class 1	Class 2	Class 3	Class 4
Class label	substance use disorder	Relatively healthy	Medically comorbid	Medically comorbid with SUD
Class size, n (%)	1293 (37.5)	1093 (31.7)	552 (16.0)	506 (14.8)
Age, mean (SD)	40.53 (12.37)	37.47 (17.36)	57.19 (11.57)	52.73 (9.97)
Female	490 (36.7)	406 (37.8)	201 (38.4)	168 (32.8)
African American	481 (36.0)	506 (47.1)	222 (42.5)	216 (42.2)
Hispanic or Latino	75 (5.6)	75 (6.9)	26 (4.9)	21 (4.1)
Insurance Medicaid	1010 (75.7)	650 (60.5)	260 (49.7)	312 (60.9)
Medicare	181 (13.6)	116 (10.8)	211 (40.3)	176 (34.4)
Self-pay	99 (7.4)	249 (23.2)	25 (4.8)	8 (1.6)
Commercial	45 (3.4)	60 (5.6)	27 (5.5)	16 (3.1)

Class 2 comprised of relatively healthy individuals with low probability for most of the comorbid conditions. We labelled this group as “relatively healthy”. The comorbid conditions with highest probability for this group are drug use disorder (28%), followed by depression (18%), alcohol use (13%), hypertension (13%), and asthma (11%). The probability of other comorbid conditions for individuals belonging to this class was near zero. This is the second largest group with 32% of the patients. Among the four classes, the proportion of patients with racial identity of Black/African American (47%), and ethnic identity of Hispanic or Latino (7%) was highest in this class. Around 38% of the patients who fell in this class were female, and 61% were covered by Medicaid. Also, 23% of patients belonging to this class were uninsured, which is the highest among the four classes. (Table 2). Class 3 included 16% of the homeless patients. The individuals in this group have very high probability of hypertension (92%), hyperlipidemia (70%), and CKD (61%), and have high probability of having diabetes (56%), depression (48%). They also have a moderate probability of heart disease (40%), and obesity (37%). The individuals in this class have low probability of having drug use disorders (27%), and alcohol use disorder (32%). We labeled this class as “medically comorbid”. Around 43% of the patients in this class were African American, and 38% were female. Class 4 included 15% of patients experiencing homelessness. The individuals in this group have very high probability of having depression (93%), drug use disorder (94%), hypertension (92%), alcohol use disorders (80%), CKD (68%), hyperlipidemia (67%) and COPD (64%). They also have high probability of having diabetes (50%), liver disease (44%), asthma (43%), and obesity (40%). We labeled this class as “medically comorbid with SUD”. Around 42% of patients in this group were African American, and 33% were female.

### Relationship of classes with time to readmission

**Fig 2:**
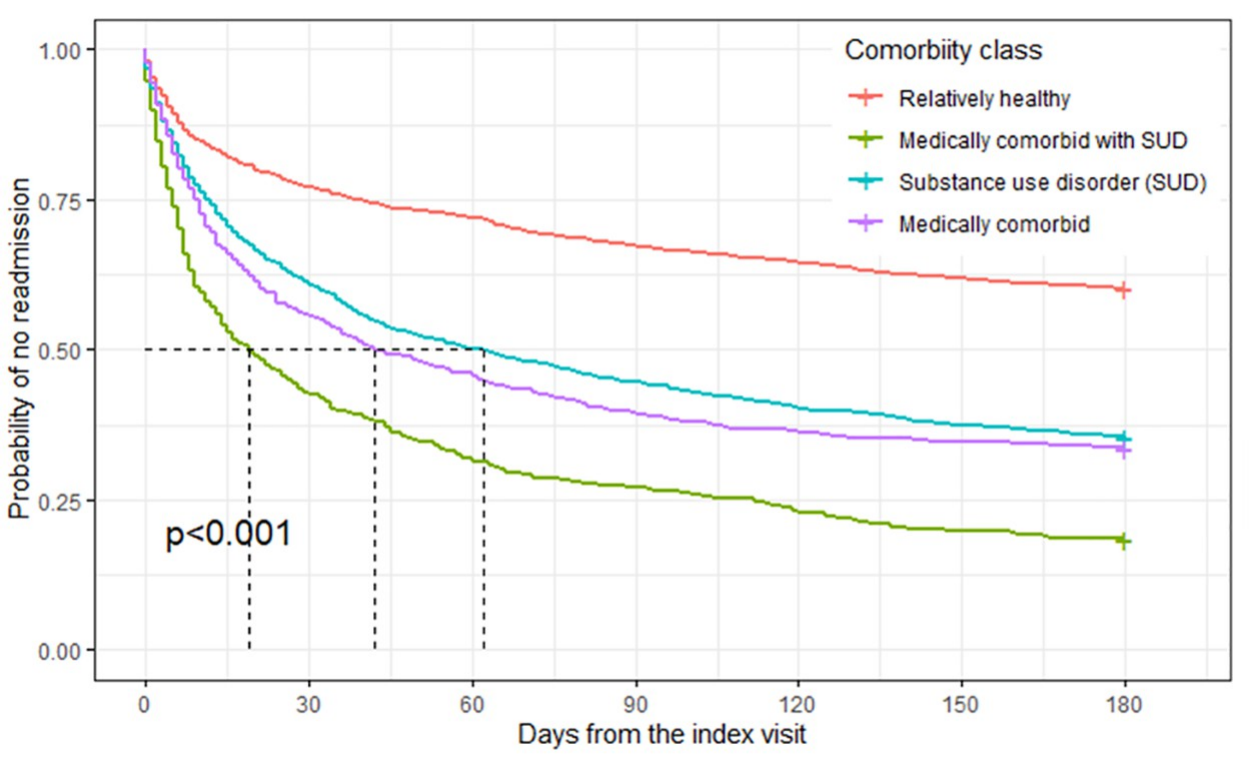
Kaplan-Meir curves of probability of no readmission against time (in days) since the index-visit.

The Kaplan-Meir curves of probability of readmission against time from the index visits were significantly different for the four classes (p<0.001), with patients in medically comorbid with SUD class having prognosis of shortest time to first readmission followed by patients in medically comorbid class, substance use disorder class, and relatively healthy class (Fig 2). The median time to readmission was 16, 44 and 60 days among the patients in medically comorbid with SUD, medically comorbid, and substance use disorder class respectively. The multivariable Cox proportional hazard model adjusted for age, sex, race, ethnicity and insurance type showed that the hazard for readmission among patients in medically comorbid with SUD class is 3.16 (CI: 2.72, 3.67) times higher than the patients in the relatively healthy class. Similarly, the hazard for readmission among patients in medically comorbid class and substance use disorder class is 2.10 (CI: 1.79, 2.47), and 1.96 (CI: 1.68, 2.14) times higher than the patients in the relatively healthy class.

## Discussion

We find that significant heterogeneity exists in the types of chronic medical conditions clustering in patients experiencing homelessness and that this heterogeneity has significant impact on the health care utilization in terms of time-to-readmission. Using latent class analysis (LCA) we identified 4 distinct comorbidity profiles of homeless patients: “relatively healthy”, “medically comorbid with SUD”, “medically comorbid”, and “substance use disorder (SUD)”. The results showed a strong clustering of drug use disorder, alcohol use disorder, and depression in two of the four classes—“substance use disorder (SUD)” and “medically comorbid with SUD”, to which more than half of the patients belong. Substance use disorder and mental health has been widely studied in homeless population both as a cause and result [3, 17–20]. While the directionality of these associations are unclear, our findings support the well documented intersectionality between substance use and housing and co-occurring mental health conditions. Policy and service delivery interventions for homelessness, especially through healthcare should consider specific assessment, support, and treatment for SUD and mental health. Multiple healthcare approaches and interventions have been practiced targeted to substance users and specifically to homeless individuals who are substance users [21]. Such interventions may include standard case management, opiate replacement therapy for opiate dependence which have been reported to be effective in reducing substance abuse and healthcare utilization [21]. The patients in “medically comorbid with SUD” class could be most challenging in terms of health care management owing to the double burden of substance use and high load of medical comorbidities like diabetes, hypertension, and COPD. The patients in this group may need multifaceted interventions including behavioral health related interventions. The Chicago Housing for Health Partnership reported 29% reduction in hospital days and a 24% reduction in emergency department visits during an 18-month follow-up among chronically ill homeless patients who received supportive housing and provision of case management [22]. Also, the patients in the ‘medically comorbid with SUD’ class have shortest time to readmission and highest hazard for readmission among the four classes making them most critical subgroup to reduce the overall healthcare utilization burden among patients experiencing homelessness. The most striking racial and ethnic disparity among homeless individuals was observed in the ‘relatively healthy’ class with African American representing 47% and Hispanic or Latino representing 7% of the individuals in this group. The higher burden of homelessness among African American and Hispanic has been well documented. The fact that this group has the youngest individuals compared to other four groups indicate a higher racial and ethnic disparity in younger homeless individuals. Though there is limited data on potential health interventions and their effectiveness for homeless young people [20], such interventions may need to focus on preventing substance misuse, and chronic homelessness. Almost one fourth of patients in this class were uninsured which could have multiple implications including accrual of medical debt arising from emergency care, and they could be less likely to receive primary care and preventive care, which in turn could result in higher acute care utilization. The relationship we showed between these comorbidity profiles and healthcare utilization in terms of time to readmission validates the clinical relevance and utility of the identified classes. Identification of these clusters among patients experiencing homelessness could help healthcare organizations better devise or adopt targeted interventions tailored to the subgroups to improve the quality of care delivered to patients experiencing homelessness. With greater financial incentives associated with reducing preventable hospital readmissions, hospital systems and organizations could better utilize often limited social resources and design different approaches toward discharge planning for higher medical needs patients experiencing homelessness. In doing so, health care providers and policy makers could not only reduce healthcare utilization deemed unnecessary, but most importantly improve the life expectancy and morbidity among those experiencing homelessness. To our knowledge, this is the first study to examine clustering of medical conditions among patients experiencing homelessness and their impact on utilization patterns. Longitudinal studies, which may use the classes and taxonomies described here, may identify causality and future opportunities for intervention. The study has several limitations. First, the study population was identified using ICD 10 code Z59.0, matching patient address in the EHR with a list of Delaware homeless shelters, and by using a social consult request field in the EHR. These approaches may not have identified all who experience homelessness or incorrectly attributed homelessness to patients. The use of patient address is dependent on an intake process at the time of encounter which may or may not have occurred accurately. The latent classes identified might be specific to the patient population that ChristianaCare serves and may not be generalizable to the overall homeless population. Additionally, this is a cross sectional study and does not capture the temporal dimension of homelessness and doesn’t provide information on how acute vs chronic homelessness would have influence these comorbidity profiles and their relationship with resource utilization. The analysis is limited to data prior to COVID-19 pandemic and additional analysis might be required to account for socio-economic impact of the pandemic in general and in homeless population.
